# Characterization and Typology of Backyard Small Pig Farms in Jipijapa, Ecuador

**DOI:** 10.3390/ani11061728

**Published:** 2021-06-09

**Authors:** Alfredo Valverde Lucio, Ana Gonzalez-Martínez, Jose Luis Alcívar Cobeña, Evangelina Rodero Serrano

**Affiliations:** 1 Faculty of Natural Sciences and Agriculture, University of the South of Manabí UNESUM, Jipijapa 130303, Manabí, Ecuador; yhonny.valverde@unesum.edu.ec; 2 Department of Animal Production, Faculty of Veterinary Sciences, University of Cordoba, 14071 Córdoba, Spain; agmartinez@uco.es; 3 Livestock Research Group, The State University of the South of Manabí, Jipijapa 130303, Manabí, Ecuador; jose.alcivar@unesum.edu.ec

**Keywords:** production system, sustainability, tropical areas, swine, non-industrial systems, small farms

## Abstract

**Simple Summary:**

The Jipijapa region in the south of Manabí Province has the maximum contribution to the pig market in Ecuador. In this region, backyard pig production is important for the economy of the small family units. The main objective of this paper was to characterize the traditional systems of Jipijapa’s small-scale pig producers and determine the farm categories according to the current characteristics of those systems for the implementation of the aid policy in the country. The study identified differences between the seven communities studied for social, productive, and local resource variables. Five types of farms were identified by multifactorial and hierarchic cluster analyses. The aspects that most contributed to the differences between those types of farms were the location, the age, the agricultural activities, the participation of women as owners of farms, the use of indigenous resources in construction, the genetics of the animals, and the food sources used. We determined that the rearing of pigs by small pig farmers of the Jipijapa region is, fundamentally, a social activity and is linked to the crops of each area.

**Abstract:**

To characterize the traditional systems of small pig producers in Jipijapa (Manabí, Ecuador) and to classify farms into representative categories, we interviewed fifty-five farmers from seven communities considering four dimensions: social, organizational, production methods, and local food resources. Multiple correspondence analyses and hierarchical clusters were carried out using the Ward method. The analysis differentiated communities based on social, productive, and local resource variables, showing three factors that accounted for 85.3% of the total variance: the socioeconomic dimension, related to the welfare of families, explained 34.4% of the variation, the care provided to animals explained 30.9%, and the management practices for the supply of food explained 20%. We identified five clusters that shared common characteristics: Group 1 included farmers from Albajacal, wage workers, and Creole pig breeders, Group 2 included farmers raising pigs under lockdown conditions, Group 3 typified traditional farms from the La Cuesta community, Group 4 included landowners, and Group 5 included professionalized farmers in Colón Alfaro. We also studied the supplied alternative food formulations made up of crop surpluses. The role of small pig farmers is a social activity linked to the location, the crops of each area, and the specific practices for the care of animals.

## 1. Introduction

In Latin America, two production systems have been identified, extensive agriculture on a commercial scale and family farming, marked by unequal access to production factors such as land, irrigation, credits, or information, which limits the capacity for their economic development [1]. Despite their small contribution to production, small producers in the tropics play a very important role in socio-economic development due to the involvement of a large number of farmers and their families [2].

In developing countries, 50% of producers are subsistence farmers whose production objective is to ensure the stability of their household consumption and access to a financial source [3]. In these systems, livestock tenure is established as a way to ensure resilience in the face of economic emergencies for the nuclear family [4]. Family farming produces more than 70% of the food consumed in Central America [5,6].

Ecuador, with 89% of agricultural production units (APU) categorized as subsistence producers [7], is one of the Latin American countries that adopted the System of Popular and Solidarity Economy (SPE), which is a dynamic and balanced relationship between society, the state, and the market that aims to guarantee production under material and immaterial conditions that enable a good living [8]. Each APU, including the family-level production, is considered an actor in the popular and solidarity economy, recognizing that this sector represents 65% of jobs and 25% of gross domestic product (GDP) [9].

The country has 828,267 APUs distributed throughout 98.27% of the 11,680,469 ha of the territory [10]. Small and medium-sized agriculture accounts for 85% of APUs and controls 20% of the land area, while commercial agriculture accounts for 15% and controls 80% of the arable soil. Despite this, family-type peasant production (small APUs) provides more than 60% of the food consumed in Ecuador [11].

Small APUs are those that do not exceed 8 ha; in Ecuador, 425,926 small APUs cover an area of 3,462,491 ha [12]. These small farms, in most cases, are livelihood farms and operate completely or in part with family labor [13].

In 2010, there were more than 100,000 APUs of backyard pig producers in Ecuador, which is equivalent to a total population of 1.4 million pigs, generating about 89,000 t/year of pork meat. On the other hand, there were 500 mechanized farms with 310,000 animals and meat production of 45,600 t [14]. However, in 2016, the scenario changed, and the pork production of semi-selected and mechanized farms increased to 84,000 t/year, while backyard pig production declined to 54,000 t/year [15]. This mechanization led to increased production of approximately 135,000 t/year [16].

The obvious abandonment of agricultural activities by small rural producers, including the rearing of backyard pigs, is worrisome, bearing in mind that these activities are important for the economy [17]. In addition, the livelihood systems host valuable zoo-genetic resources that need to be conserved because they have evolved to adapt to adverse environments, and there are situations in which biodiversity is being lost [18]. 

Reasons for this abandonment include the following: resistance to adherence to regulations of health control by the Ministry of Agriculture and Livestock, which seeks to ensure the safety of the meat consumed [16], the high price of inputs, the non-appreciation of the quality of carcasses produced by small rural producers, limited technical assistance, the application of new technologies [19], and the exodus from the countryside to the city [20]. Pig meat production fell by 16% and brought about changes in the production systems [12]. In 18 years, the breeding of commercial breed pigs increased from 2% to 53% and their cross by about 21%, while Creole pork fell from 78% to 25%.

Manabí, the third-largest province in the country, led in national swine production until 2000, but currently ranks third, with a 31% decrease in production [12], as it has experienced a decline in APUs more than other areas. Pig production in Jipijapa (the southern microregion of Manabí) was 15,330 pigs until 2010, showing an increase of 61% compared to the agricultural census of 2000 [21]. Most of the producers in the region are peasants who engage in backyard production and commonly employ mixed pigs or Creole pigs from the Iberian breed introduced during the Spanish conquest [22], which are preferred for their rusticity and environmental and food adaptation. Feeding of backyard pigs ensures financial returns to the farmers from their reproduction and meat production, despite their unbalanced nutritional management dependent on local agricultural by-products [23].

There are no organizational structures around the production of pigs—the producer individually decides how many pigs to raise and how to market them. Farms range from one to five pigs, although there is a small group of producers who raise between ten and twenty pigs, and many APUs have ceased swine activity. In 2014, this heterogeneity motivated a study to characterize the socio-productive systems of backyard pigs in Jipijapa, specifically concerning the coffee production [24].

The diversity of systems in swine production may be determined by concomitant agricultural activities, by their geographical location, or by complex combinations of factors. Therefore, the characterization of this sector implies a search for typologies or group farms according to their similarities [25,26]. This makes it necessary to apply typification methodologies with cluster analysis of economic and social structural variables [27]. In this way, we could identify specific productive strategies that can improve the opportunities of the studied areas [28], prioritizing support, proposing specific measures, innovations, and policies for each identified category [25,29,30].

In this work, an up-to-date characterization of family backyard pig production systems in the rural sectors of the Jipijapa canton was performed to enhance the knowledge regarding the capacity of backyard pig breeders and identify the prevailing systems and factors that limit production and sustainability. This information will permit the identification of their relationship with the agricultural activity, the sociocultural and organizational aspects of this population segment, and the economy of the sector. Based on these results, recommendations can be made to promote this sector of the SPE and improve its impact at the national level. It allows to prevent the disappearance of these sustainable livelihood systems and facilitate the settlement of families in agricultural territories [31].

## 2. Materials and Methods

### 2.1. Study Area and Data Origin

**Figure 1:**
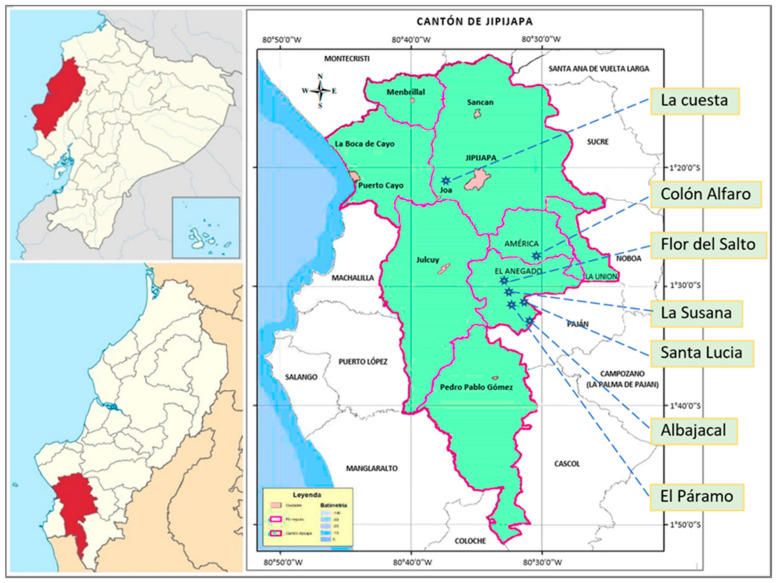
Geographic location of sampling zones.

The study was carried out in the southern microregion of Manabí Province, Jipijapa canton, and El Anegado parish (Figure 1), by the high percentage of the rural population (60%). This area is geographically located between 1′10′ and 1′47′ south latitude and 80′25′ and 80′52′ west longitude, with a local steppe climate considered BSh, a variant of the dry subtropical and warm semi-arid climate, with an average annual temperature of 23.7 °C and an average annual rainfall of 537 mm [32]. The population was 71,083 inhabitants as of 2010, with the population growing by 8.9% in the last nine years. The territorial area is 1567.45 km^2^, surrounded by an isolated and irregular massif mountain system, ending at the Colonche mountain range in the Jipijapa valley [33].

The characterization was carried out in three aspects:

(a) Socioeconomic, which allowed us to identify age, schooling, specialized training, membership in associations, basic services, amount of time dedicated to raising pigs, animal health, waste management, and perception of aid from development bodies.
(b) Productive, to characterize the operating system, breeds used, means of production, materials of construction, control of productive records and planning, type of animals slaughtered, and production levels.
(c) The local resources available to producers for the rearing of animals, accounting for the types of local food inputs provided to the pigs, their origin, the equipment, and the ways of preparing or supplying food.

For this study, a static survey of 30 close-ended questions was designed, as well as recording the community in which each producer was located. The survey collected information on each area, with 12 socioeconomic, 12 productive, and 6 local resource variables. These qualitative variables were all of a nominal scale, ranging from 2 to 16 categories of responses. The productive aspect was supplemented by seven quantitative variables about the production levels related to the number, price, age, and weight of the pigs.

The survey was carried out with 55 producers, among the approximately 150 who live in the studied region according to the Territorial Ordering Plan (TOP). There were no official public registers or organizations of pig farmers. The survey was then completed following the “snowball” methodology [34], which begins by locating certain individuals, leading to others, etc., until achieving a sufficiently high and representative sample number. In this way, all producers who were willing to provide information were interviewed.

The surveys were conducted in seven communities, the majority in the EL Anegado parish, in the rural Jipijapa region, which is considered the cantonal head of Manabí. The TOP of El Anegado parish identified the communities that were most representative of the geography and population, and as a result, the work began in the Colón Alfaro community and then continued according to the method described, resulting in the following sample: Colón Alfaro, 10 producers; Albajacal, 10 producers; La Susana, 6 producers; Flor del Salto, 6 producers; La Cuesta, 13 producers; Santa Lucia, 5 producers; El Páramo, 5 producers. In these communities, with their varied climatic characteristics, relief, and agricultural production, the provision of inputs for animals and the production of pigs throughout the year are guaranteed.

### 2.2. Statistical Analysis

For the initial characterization of Jipijapa’s backyard pig farms with data from the 55 surveys, the absolute and relative frequencies in % and the descriptive average and variation statistics related to production levels were calculated. 

In the second phase, multiple correspondence analysis (MCA) was used to analyze the relationships between categories of variables and to identify the dimensions defined by the associations between them. The percentage of variance explained the definition of factors. MCA was performed only with those variables that had the greatest variation and were not correlated with the others. For MCA, quantitative variables had to be transformed into bivariate ordinal variables according to Diaz and Garrido [35]. 

Subsequently, with the same variables as those used in MCA, a sequential analysis of hierarchical conglomerates was conducted to group the livestock and identify typologies. The solution for proper classification of all livestock was found using Ward’s method and the Euclidean distance, which maximizes the homogeneity within groups and the greatest differences between groups [36]. For the definition of the characteristics of each identified cluster, the characteristics corresponding to variables of more than one option were expressed in bivariate mode in response to yes/no. The comparison between clusters was performed using ANOVA for quantitative variables and by Duncan tests for qualitative variables at the *p* < 0.05 level. For all analyses, we used SPSS v.25 statistical software.

## 3. Results

### 3.1. Characteristics of Jipijapa’s Small Pig Farms

Table 1, Table 2, Table 3 and Table 4 present the characteristics of backyard pig production in the three areas studied in the Ecuadorian Jipijapa region, with more farms identified in the community of La Cuesta, which had 13 producers.

The age range of the farmers was very wide, from 17 to 88 years, with an average of 52.31 ± 2.36 years. The results obtained from the socioeconomic or structural field also showed that, out of the 55 producers, 15 were women, 45.5% only finished primary school, and only 7.3% had a higher education, but not complete (Table 1). The owners of the farms were mostly the heads of households (70.9%), although the work of animal care was mainly carried out by women and children (43.6%). 

Labor occupations were shared with agricultural activities on the farms (36.4%) or with day labor on nearby farms (38.2%), but they rarely involved exclusive pig rearing (1.8%). Most pig producers of Jipijapa had only electricity among the basic services (60%). In 21% of cases, they lacked electricity but had piped water. The most common source of water designated for pigs among pig farmers in Jipijapa was welling (72.7%). 

Regarding garbage management, only 21.8% of farms had a collection service, and the majority (49.1%) buried their waste. Only 9% of producers had all these services, and none had access to the internet. Regarding socio-organizational aspects, 32.7% belonged to an organization, while only 9.1% benefited from a project or grant from the Ecuadorian state. Regarding training, public entities did not influence the sector for training. Although the vast majority (94.5%) would like to receive specialized training courses, only 14.5% of those surveyed had received them.

In terms of production, the results show an intensive swine farming system (49.91%), or mixed (43.6%), with very scarce direct use of grazing resources (Table 2). The most common diseases in livestock were respiratory diseases (56.4%), although 36.4% reported the presence of multiple conditions, such as parasitism, gastrointestinal, and respiratory tract infections. Most of the producers (43.69%) know and carry out basic veterinary practices, such as the application of vitamins, deworming, and male castration. 

Traditional pigsties are built to take advantage of various natural materials, such as cane, wood, and cadi leaves for the ceiling and a cement floor, and 54.55% of the pigsties were of this type. Other pigsties used only one of these elements, i.e., 29.09% used cane, 9.9% used wood, and only 7.27% had concrete walls and flooring. 

Regarding the breeds of pigs that were selected, local genetic resources were found in all communities studied, with 61.82% of producers producing Creole pigs. Studies could be conducted to characterize and differentiate them fully. To reduce the cost of piglets, producers purchase pigs from other producers who do not control crossbreeds with other breeds. Therefore, 14.55% raised mixed-breed pigs, and very few had commercial pigs, such as Pietrain, Landrace, and Duroc breeds. 

Only 23% of producers offered water to their animals ad libitum with automatic drinkers, while the majority offered water twice a day, in the morning and the afternoon (69%). The same system was used for the food supply (90.91%), using rustic feeders made with plastic containers, car tire rubber, or cement. 

The local producers commonly did not register their productive agricultural activities (85.45%), and those who did so merely checked dates without using a well-crafted technical document. This situation is similar regarding the food register: 87.27% did not keep control over the costs of food given to pigs, especially those who supplemented the food requirements with agricultural products that they harvested on their own plantations. Producers justified the rearing of pigs only for self-consumption (21.82%), although 52.73% did so for economic reasons.

At the production level, the parameters differed greatly between producers (Table 3). The number of pigs bred was 4.44 ± 0.55 on average. Producers commonly raised one or two pigs, and others reached up to twenty. However, the probability of finding producers raising more than 10 pigs was less than 10%. The pigs’ age of when fattening started also differed greatly between producers, ranging between 1.5 and 5 months, with an average of 2.47 ± 0.13 months. They were slaughtered at 6 months in some farms and later in others, reaching the age of completion at 18 months. 

The weight of the piglets at the onset of fattening was 27.36 ± 2.135 lb on average, being associated with different ages: some started at 10 lb, and others at 70 lb. The average final weight was 115.91 ± 3.96 lb, with a range between 70 and 200 lb. All of these differences are due to different cultures in the production process between communities (management, knowledge, types of food, and ways to supply it), as well as the breeds used for production. The prices received for the product were also quite heterogeneous, with an average of 1.45 ± 0.03 USD/lb for live animals and 2.57 ± 0.04 USD/lb for slaughtered animals.

Regarding the local resources, 60% of pig farmers in El Anegado region cultivated fodder to feed their animals and acquired the remaining products from plantations or communities (Table 4). Food alternatives specific to the pig farming sector, including the most abundant agricultural products in the area, were used by 54.55% of the producers. In any case, balanced food was always combined with these food alternatives, which are of great interest in the pig production system of this region. 

In the relationship with food alternatives, 16 combinations were identified in which agricultural products were combined with cooking waste. The most frequently used products included maize, banana, pumpkin, cassava (yucca), ivory palm, and rice powder. Producers did not have a mill to produce flour and feed formulation equipment, and half (50.90%) prepared cut-up food, including cooked food (34.5%), especially banana.

### 3.2. Multiple Correspondence Analysis (MCA) for Variation of Small Family Farms in Jipijapa

A high percentage of the variation (85.5%) could be explained by three factors or dimensions (Table 5). Factor 1 (F1), accounting for 34.4% of the variance, represented the operational aspects of swine production in the rural sectors studied, as it mainly related to the basic resources and infrastructures for the welfare of pigs and producers’ families. The variables involved in this dimension were basic services, water sources, waste management, pig breeds, and sale price at slaughter.

The second factor (F2), explaining 30.89% of the variance, referred to the relative importance of the care provided to animals and involved the construction materials of the pigsty, duration of fattening, and food of the farm, in addition to the various occupations and practices of planning and registration, which also showed importance. The third factor (F3), explaining more than 20% of the variance, related to the dimension of food management, in which the most important variables were those related to supply practices, involving time and how pigs were supplied the alternative food, as well as the age of onset of fattening.

### 3.3. Cluster Analysis for the Typology of Small Pig Farms in Jipijapa 

**Figure 2:**
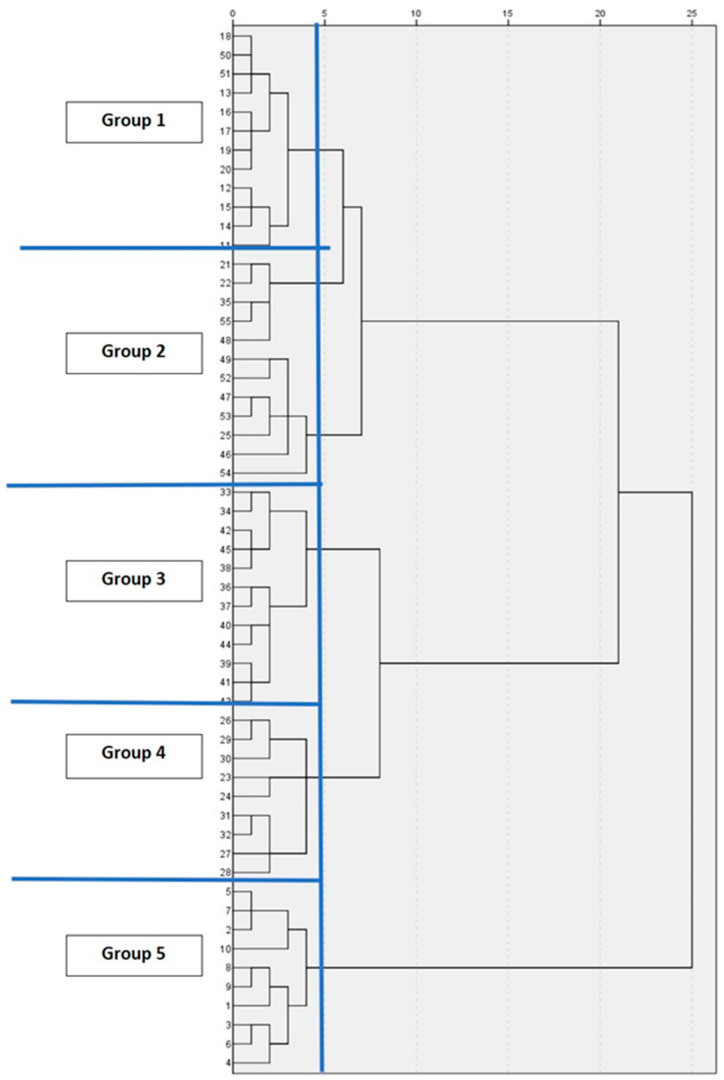
Dendrogram for clustering and identification of groups of small pig farms in Jipijapa.

The cluster analysis established five groups to identify farm categories: groups 1–3 (G1, G2, G3) with 12 farmers in each group, group 4 (G4) with 9 farmers, and group 5 (G5) with 10 farmers (Figure 2). 

**Figure 3:**
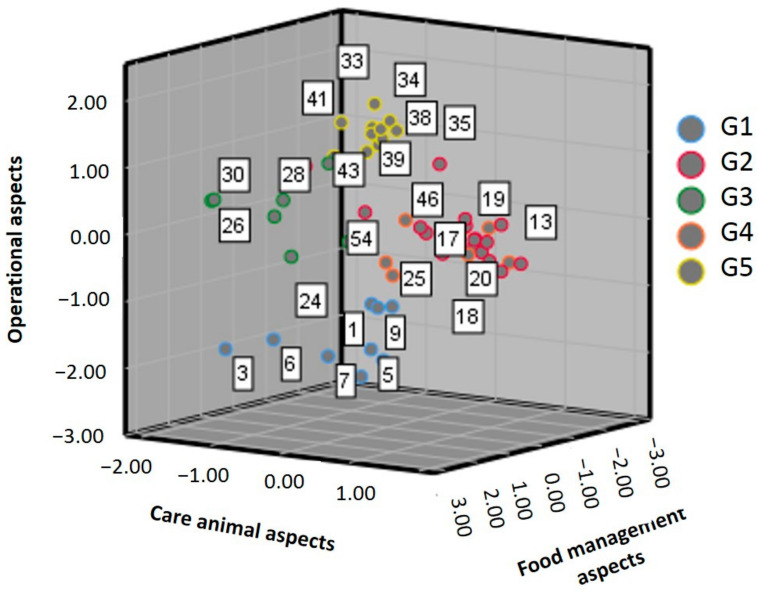
Cluster interaction and MCA dimensions.

Figure 3 shows the distribution of farms in the rural sectors of Jipijapa considering the three dimensions corresponding to the MCA factors and contrasted with the five identified groups or clusters, which showed that backyard pig farming was found to be primarily an operational activity, linked to the families’ welfare and to the management of the production system, where food times and forms were the outstanding aspects in the breeding of pigs. The greatest homogeneity linked with the three factors was found in G2 and G5 groups, while G1, G3, and G4 groups had a higher dispersion of data, G3 and G5 were diverted to the social welfare dimension (F1), G1 was positioned toward the dimension of caring for pigs, and G2 and G4 were more geared toward the factor explaining food and fattening practices (F3). The last factor has crucial importance to the sustainable pig production systems of Jipijapa by taking advantage of the remains of agricultural crops in the area to fatten the pigs.

### 3.4. Characteristics of the Types of Family Pig Farms in Jipijapa

The definition of types showed a clear effect (*p* < 0.001) of geographic location, having G5 and G3 group breeders only in the communities of La Cuesta and Colón de Alfaro, respectively (Table 6).

The characteristics of the identified groups according to the three areas analyzed are shown in Table 7.

#### 3.4.1. Group 1: Albajacal Wage Worker Creole Pig Breeders

The first group (G1) characterized 12 older pig farmers (66.67 ± 14.08 years) mainly from the community of Albajacal, who worked as day laborers (83.33%) and once belonged to an organization (66%). Most had only completed primary school, drank well water, and lacked waste collection, but had electricity. Most (66.7%) employed the system of locking in cane pigsty by 90% to raise about three castrated pigs of the Creole breed destined for self-consumption, which were under the care of the wife and children. 

These pig farmers started pork fattening very early, at 2.25 months on average, and with very low weight (15.42 ± 2.57 lb), so the pigs needed to be fattened for a long time, up to the age of 12.83 months on average, to take them to an average final weight of 107.50 lb. Agricultural products were purchased from other farms. They did not typically use balanced food, and they often took advantage of waste from other farms and kitchen waste. A total of 67% cooked the food alternatives, and the rest simply chopped the food. They used an average of six products in the composition: together with maize and cassava, they usually added banana and pumpkin, among other local products. 

#### 3.4.2. Group 2: Intensive-Breeding Pigs 

The second category (G2) referred to 12 farms distributed among the communities of La Susana, Santa Lucia, and Paramo. Half of the heads of farms were women who had completed primary school (83.33%), and the average age was 51.75 ± 17.62 years. They bred more under lockdown conditions (75%) and for sale (58.40%) and did not depend on state subsidies. They were not engaged in agriculture, although 50% sowed food for livestock, which were Creole pigs in 66.67% of cases. 

They did not record or plan their production, and their properties were of traditional construction, housing an average of five pigs that entered at a somewhat older age (19.17 ± 7.64 lb) and finished slightly later, at 115.83 ± 21.93 lb on average. Although they had running water, it was not freely available to the animals, and was instead provided with the food rations once or twice a day. Approximately 67% cooked food alternatives, which were made up of combinations of five products, including bananas along with corn and cassava as a constant element. 

#### 3.4.3. Group 3: Traditional Type Producers in the La Cuesta Community

The third category (G3), located exclusively in the community of La Cuesta, involved the participation of 12 producers. They were mostly men with very different ages, between 20 and 75 years old, and 75% had no education or only completed primary school. They were not associated and had not received state aid. They lacked basic running water and waste collection services, and only 41.67% had electricity. The production system was mixed, always taking the animals and providing them traditional pigsties available in almost all cases (95%). 

These farmers bred 2 to 15 pigs, mostly of introduced breeds (60%), and they considered production as more of a business, since they reported that they did not raise them for their own consumption, although they did not keep records or castrate and start fattening when the pigs reached three months and weighed more than 40 lb. They did not grow food, they bought foods composed of five alternatives on average that were always served without cooking, cut up only, and were typically supplemented with feed (75%). With this, the animals quickly (before five months) reached completion weight (110.83 ± 23.91 lb).

#### 3.4.4. Group 4: Farmers Who Owned Their farm

The fourth category (G4) located mainly in Flor del Salto with some producers in the community of La Susana, included nine producers, mostly men (89%), whose average age was 54.33 ± 19.11 years. A total of 90% had only completed primary school, and all were married and delegated animal care to their wife and children. All were farmers, mainly on the farm itself (89%), who claimed to breed mainly as a business. They had well water and electric power; however, few had a garbage collection service (33%). Among them, 55% were members of an organization but had not received support from the state. 

All of them used a mixed production system, so the animals, mostly Creole (67%), had the opportunity to go out to graze and be housed in traditional type sties (67%), which, in some cases, were more technical, as 45% had permanent water supply facilities. They did not record or plan production (67%), and many did not usually castrate the animals (45%). Only 67% performed deworming. They all grew food for livestock but also fed the animals with cooking waste (89%) and balanced supplements (67.7%). 

These farmers used at least two food alternatives consisting of about five components, among which the use of ivory palm stands out (67%). Additionally, they are characterized by the absence of pumpkin and banana. Food was typically cooked (78%), and fattening started very early, before two months, when the pigs weighed barely 18 lb, and took an average of seven months to finish at a weight of 115.90 ± 29.19 lb. Despite the small size of the animals, they fetched good prices, both live and slaughtered (2.89 ± 2.02 USD/lb).

#### 3.4.5. Group 5: Professional Farmers of Colón Alfaro 

The fifth category (G5), fully located in the community of Colón Alfaro, was made up of 10 producers, mostly men (80%), who left the rearing of pigs to their wives and children (90%). They had very different ages, ranging from 17 to 69 years, and had the highest level of schooling, as almost 70% had finished high school, 30% had been professionally trained, and 10% had higher education. Almost all were self-employed farmers (90%), and only one worked as a day laborer. All had basic services (electricity, piped drinking water, and garbage collection), were not organized, and had not received government support. 

These famers raised very few pigs: half raised fewer than five Creole pigs and half-breeds, and all did so in an intensified and planned way, keeping records of production and practicing deworming. The slings were made of cement and wood, they started fattening at an average age of just two months but at a good weight (29.49 ± 6.85 L), and finished them after about seven months at an average final weight of less than 100 L; therefore, sales prices were very low (1.23 and 2.27 USD/lb on average for live and farmed animals, respectively). 

There were 60% who reported raising animals for consumption only. They did not use commercial foods, and half grew food for livestock and added kitchen waste to the food alternatives, using only two or three products, among which banana was usually present (80%). The food was usually fed to the pigs without cooking (70%).

## 4. Discussion

The high variability between types of producers and forms of livestock management found for family production systems with pigs in Jipijapa coincides with what was found by other authors in tropical systems [37]. It is appreciable that the rural sectors of Ecuador have limitations at the level of basic services [38]. The dimension related to infrastructure, represented by the variables of basic services, water sources, and waste management, which are key elements for the operational and the welfare of families’ point of view, was pivotal for the classification obtained in this work. These factors were previously shown to be directly related to the profitability of production [39].

We found that small pig producers in Jipijapa intended to sell the animals, and a lower percentage bred them only for self-sufficiency (36.6%). Similarly, Solís et al. [40] found a lower percentage (25%) of Ecuadorian goat producers in Santa Elena who only bred the animals to meet their basic needs. 

The pig farmers of Jipijapa were mostly tenants or were employed as wage workers [7], which was less pronounced in the Colon Alfaro and Flor del Salto (G5 and G4) community groups that include the majority of landowners. Leasing is often preferred over land ownership due to the high capital requirements for land acquisition and the high risk of investment [41]. Our results show that the establishment of aid for the acquisition of land would be particularly important to develop investments and stabilize the Albajacal (G1) farmers to the territory [31].

The age and education level of the producers are key elements in farmers’ ability to adapt to socioeconomic changes, which is directly related to the ability to implement production strategies on farms [42,43]. Young people tend to have a higher level of education and have been seen to achieve better productivity levels and profitability rates. Therefore, they are less dependent on subsidies [44] and appear to have a greater inclination toward organic production activities [45]. The average age of the small pig farmers in Jipijapa was 53.31 ± 17.52 years, and the community of Albajacal had the oldest population, which proved to be a determining factor in the organization of the groups we identified. Our data coincides with the goat systems in Santa Elena (Ecuador) reported by Solis et al. [40]. However, regarding the producers’ training level, our result in Jipijapa were more favorable than Santa Elena (72.7% vs. 61% of producers who have at least completed primary school, respectively). 

The agricultural activity that prevails in the canton of Jipijapa are the monocultures of banana, coffee, corn, cassava, etc., which were highly dependent on the frequent changes in commercial demands and agricultural policies occurring in Ecuador since the late 1990’s. This has resulted in peasant families having to seek forms of resilience and diversified production modes and farm work [46]. The backyard pig production systems in the studied communities of the Jipijapa canton are part of the organization of the agricultural farm to cover the needs of the families. Although the sale of 3–5 pigs per year does not constitute a relevant commercial activity, this contributes to the family economy, which was found to be complemented by the self-sufficiency in 52% of the interviewees. This point of view is essential to understand and explain the reported characteristics of the agroecosystems of the backyard pig in Jipijapa.

Thus, generally (84%), but more especially in Group 4, the care of the pigs remains in the hands of women and children (100%), while men move to perform agricultural work on farms located in the vicinity of the housing, or as laborers on other farms nearby (3–5 km away) during the planting or harvest seasons. Our results agree with those found in other agricultural areas with similar conditions, such as Río Blanco—Nicaragua (98.52%) [47] or dairy cattle systems in the Sierra Norte of Ecuador [31]. However, they differ from other agricultural systems in rainfed areas in Ecuador, such as goat farms in Santa Elena (37%), where management and grazing systems are extensive and are not integrated with the agricultural work.

The economic processes at the territorial level throughout Latin America have led to a high feminization of agriculture [48,49]. In the case of Ecuador, Vascónez et al. [4] explained that women take the leading role due to the men’s need to carry out wage-earning activities in the cities. This is not the case in Jipijapa, where the city of San Lorenzo is small (approximately 48,000 inhabitants) and does not offer great job opportunities to people living in the agricultural area. Our results suggest the need to carry out plans to bring technical training to the communities from the G1, G2, and G3 groups, especially aimed at women and children raising the pigs.

Da Silva [50] noted the farm facilities as another important factor for pig production and fattening. The characteristics of the backyard pigs in Jipijapa also contributed to the typification of the groups; the producers of La Cuesta, making up G3 group, used traditionally built pigsties with cane as the most used material for the walls and straw (cady) or zinc sheets for the roof. In Brazil, traditional accommodations are used by 50% of Bisaro pig producers [51]. Nath et al. [37] provided a similar description of traditional piggeries, indicating that they are made from bamboo and wood, as these materials are available in the vicinity, which implies reduced labor costs but does not strictly respect the standards of health and environmental hygiene [52].

With exception of backyard pig farmers from the G5 group, production planning and recording practices are not usually carried out (only by 29.09% and 12.73% of producers, respectively). Beyli et al. [53] indicated that poor planning affects the sustainability of Ecuador’s production system, increasing production costs, and may even dilute or, in some cases, eliminate profitability. In this sense, Acero [54] and Cattaneo et al. [55] also indicated that planning provides several advantages: the provision of work, the application of good practices, a reduction in social problems, an assortment of various nutrients, the management of feeding schedules and bedding, and reproductive adaptation. Therefore, it is imperative to keep records of the chosen swine production system regarding not only productive data but also planning tasks and operations that require training and technical advice [56]. This would be especially important in the G2 group, where only 8.33% of farmers plan the animals’ reproduction.

The variables of the third dimension (F3) named as “food supply practices” included the dedication to food and water supply to pigs (attention to feeder and drinkers, etc.). They are relevant due to their contribution to reducing the risk of disease transmission to the consumer, ensuring the health quality of the final product [57,58]. La Cuesta farmers (G3) do not have running water, so the animals’ water is rationed in all cases, with grazing being the main feeding system (only 25% of supplementation). Our results have shown the need to improve accommodation and practices to provide them with food and water in the G3 group.

Conservation of local zoo-genetic resources is valuable in subsistence systems as they are biodiversity reservoirs and have evolved in adapting to their environment [18]. In all the studied rural communities, Creole pigs were produced (61.82% of total cases). However, because of the cost of piglets, pigs are often acquired from other producers who do not control crosses with other breeds (14.55%). In another region of Ecuador (El Oro Province), the situation with respect to Creole breeds was similar: 53% of farms had Creole pigs and 37% had crossbreeds [59]. At the global level, similar situations are also observed in the family porciculture of Mexico City [60] and in Baraguá–Cuba [61], which are also characterized by the breeding of Creole pigs or combinations with improved crossbreeds.

In this work, the genetics of the pigs produced has been a decisive aspect for defining the category of G1 (Alba-jacal producers) being pure Creole pig breeders. The meat of the Creole pigs is not appreciated by the consumers of Jipijapa, due to its higher amount of fat. For this reason, the price for the sale of Creole pigs is lower than that of pigs of other breeds. The Albajacal community could be one of the last niches for the conservation of the endangered Creole pigs of Ecuador. The preservation of this local zoo-genetic resource would require international or national aids that allow the implementation of an in situ conservation program in Albajacal, the creation of small meat processing industries, and the promotion of the differentiated quality of the products of Creole pork produced in the area. In Ecuador, an adult pig is sold for USD 100 to 150, and the differences could be related to the added value provided by the health guarantee of the operation, that in 10% of cases is carried out at the home level and without veterinary inspection [16]. In our study, the established groups showed a large difference in the sale price obtained for slaughtered pork, reaching prices in G4 of 2.89 ± 0.22 USD/lb on average, which do not appear to be related to the prices obtained per sale alive. These results suggest that consumers prefer meat from younger pigs. This is the case of the G4 group farmers, who sacrificed their animals at 8.44 ± 1.74 months and sold them at 2.89 ± 0.22 USD/lb, in comparison with the G1 group, who received 2.50 USD/lb at a sacrifice average age of 12.83 ± 2.48 months. The duration of fattening was 9.3 months on average. However, the fattening cycle in most cases ended at 18 months, which is attributed to the supply with food only from the area without considering the nutritional content [37]. Hence, detailed economic studies should be developed to assess the production costs of each of the feeding strategies that different groups are currently performing in order to determine the optimal moments to start the fattening period and the slaughter age.

In this work, all producers used alternative formulations based on local agricultural food and kitchen waste to reduce the cost of the pigs’ feeding. This finding agrees with other studies in tropical and subtropical countries [2,61,62,63]. However, although Paixao et al. [51], in Brazil, reported that 94.7% of producers completed feeding with crops from the farm itself, grazing was present in 40% of cases, while in our study, it was only present in 28% of cases.

The most frequently used products (corn, cassava, banana, pumpkin, tagua, and rice powder) and the combinations in which they were supplied in the feeding of backyard pigs in Jipijapa were found to be differentiating factors among the five groups given the dependence on crops in each zone and the availability or harvest time. In this way, banana and pumpkin were not included in the diets by producers in G4 and G5, as these foods are not usually grown in those communities (Colón Alfaro, Flor del Salto, and Susana), whereas these products were commonly used by most of the G1 group producers located in Albajacal.

Only 45% of producers interviewed used commercial food. Nevertheless, this was always combined with their own productions’ by-products, commonly made with home-grown corn and by-products such as rice powder, and to a lesser extent, soybean paste due to the high cost. In 50.9% of cases, soybean paste was supplied raw, without caution for anti-nutrients (e.g., cyanoglucosides and tannins), which usually resulted in a state of pigs’ chronic intoxication [64], as cited in [65]. Likewise, the green bananas used by the first three groups contain large amounts of free tannins, which produce an astringent flavor that limits their voluntary consumption and digestibility [66]. The bananas’ nutritional value would be improved through physical or chemical treatment to be used in pigs’ feeding, as was demonstrated with potatoes by González-Torres et al. [67]. Hence, in order to have effective, economical, and safe local alternatives to commercial feeds, the potential use of local Jipijapa products should be investigated considering the optimal formulation, acceptability, the best mode of supply, and the effects on pork growth and health. From the sustainability point of view, these practices will help in reducing crops and kitchen waste and to make the most of food resources, thus contributing to the circular economy. In low-income countries with high levels of food insecurity, reducing food loss can have a positive impact on food and environmental exploitation. As reported by the FAO [68], Ecuador together with Peru have the greatest diversity of agricultural foods but register the least amounts of fruits, vegetables, cereals, legumes, and roots waste and by-products. Their use as fodder for animals could be contributing to these good results. 

Our results, in comparison with those reported by García-Martínez et al. [3], show a very low and varied level of innovation between regions, and that small producers of backyard pigs in Jipijapa intend to produce enough to ensure food for their households and a stable source of income. Appropriate incentives and plans ensure access to basic services, and increased training, are needed to improve producing families’ lives. Modernization (in terms of technology, foods, and breeds), farm infrastructure amelioration, and livestock management practices have proven to be essential factors to increase production, thus contributing to improving family income for Ecuador’s producers [1,69].

## 5. Conclusions

The research determined the socio-productive importance of backyard pig farming for families in the rural sector of Jipijapa canton and characterized the production systems. The sampling and survey and the approach methodology used to determine the diversity of small family-type farms with backyard pigs were adequate to record the existing variability and to identify groupings.

Depending on the location and socioeconomic, management, and feeding practices analyzed, we investigated the main factors explaining variations among five types of farms and identified their characteristics. This can be used as a tool to help producers and institutions determine local potentialities, to identify weaknesses, and to establish the most appropriate innovations for each type.

This also sheds light on the role played by small family farms in Jipijapa in swine production and the interest in driving efforts toward the agro–ecological transition. The linkage of small producers to agricultural activities allows them to carry out sustainable production based on their feeding strategies with the use of by-products of crops and cooking waste that, together with the use of Creole pigs, allows a reduction in the use of external inputs.

The results identified the structure of the sector and the critical points and invite reflection and sustainable solutions to improve the family production of the backyard breeding of pigs in Jipijapa. 

We detected a need to analyze the toxicity and productive efficacy of the formulations used and to characterize the genetic resources that are bred.

## Figures and Tables

**Table 1:** Characteristics of socio-economic or structural confines of small pig producers of communities of El Anegado parish, Jipijapa (N = 55).

Variable	Class	Absolute Frequency	Relative Frequency (%)	MCA *
Community	Colón Alfaro	10	18.2	*
	Albajacal	10	18.2	
	La Susana	6	10.9	
	Flor del Salto	6	10.9	
	La Cuesta	13	23.6	
	Santa Lucia	5	9.1	
	El Páramo	5	9.1	
Schooling	Incomplete primary	10	18.2	*
	Complete primary	25	45.5	
	Incomplete secondary	2	3.6	
	Complete secondary	13	23.6	
	Incomplete higher	4	7.3	
	Professional	1	1.8	
Sex	Male	39	70.9	
	Female	16	29.1	
Family dedication	Husband	9	16.4	
	Wife	10	18.2	
	Children	12	21.8	
	Wife and children	24	43.6	
Occupation	Professional	1	1.8	*
	Laborer	20	36.4	
	Farmer	21	38.2	
	Housewife	10	18.2	
	Student	3	5.5	
Basic services	Pipe water	9	16.4	*
	Electric energy	33	60.0	
	Pipe water and electric energy	8	14.5	
	All	5	9.1	
Water supply	Pipe water	12	21.8	*
	Well water	40	72.7	
	River water	3	5.5	
Waste control	Garbage truck	12	21.8	*
	Burn	12	21.8	
	Bury	27	49.1	
	Solar dump	4	7.3	
Breeding by	Sell	26	47.3	*
	Self-consumption	20	36.4	
	Both	9	16.4	
Breeding reason	Food	12	21.8	
	Economy	29	52.7	
	Both	14	25.4	
Belongs to an organization ¹	Yes/No	18	32.7	
Part of an organized project ¹	Yes/No	5	9.1	
Received training courses ¹	Yes/No	8	14.5	
Would like to be trained ¹	Yes/No	52	94.0	

^1^ Represents the number of affirmative answers. * Variables used for multiple correspondence analysis (MCA).

**Table 2:** Characteristics of the productive scope of small pig producers in communities of El Anegado parish, Jipijapa (N = 55).

Variable	Class	Absolute Frequency	Relative Frequency (%)	MCA *
Production system	Intensive	27	49.1	*
	Extensive	4	7.3	
	Mixed	24	43.6	
Preventative practices	Castration	5	9.1	
	Deworming	6	10.9	
	Vitamins and deworming	20	36.4	
	All	24	43.6	
Pigsty construction material	Cement	4	7.3	*
	Wood	5	9.1	
	Cane	16	29.1	
	Traditional	30	54.5	
Pig breeds	Creole	34	61.8	*
	Cross-bred	8	14.5	
	Pietrain	9	16.4	
	Duroc	3	5.5	
	Landrace	1	1.8	
Water supply ^2^	Morning	4	7.3	*
	Morning and afternoon	38	69.1	
	All day	13	23.6	
Food supply ^2^	Morning and afternoon	50	90.9	*
	All day	5	9.1	
Breeding time record ¹	Yes/No	8	14.5	*
Food costs record ¹	Yes/No	7	12.7	*
Common diseases	Parasitism	2	3.6	*
	Respiratory	31	56.4	
	Diarrhea	1	1.8	
	All	20	36.4	
	Other	1	1.8	
Plan production ^1^^,^^3^	Yes/No	16	29.09	*
Record productioncosts ^1^	Yes/No	7	12.73	*

^1^ Represents the number of an affirmative answers, ^2^ they were combined to form a practical ordinal supply variable, and ^3^ combined to form nominal variable planning and recording practices. * Variables chosen for multiple correspondence analysis (MCA).

**Table 3:** Descriptive statistics of pig production levels in El Anegado parish, Jipijapa (N = 55).

Variables	Median	Standard Deviation	Standard Error	Minimum	Maximum	MCA *
Number of pigs	4.44	4.11	0.55	1.00	20.00	
Age at the start of fattening (months)	2.47	1.00	0.13	1.00	5.00	
Final fattening age (months)	9.53	2.69	0.36	6.00	18.00	
Fattening start weight (lb)	27.36	15.84	2.14	10.00	70.00	*
Final fattened weight (lb)	115.91	29.19	3.94	70.00	200.00	*
Live sale price (USD/lb)	1.45	0.22	0.03	1.20	2.50	
Slaughter sale price (USD/lb)	2.57	0.28	0.04	1.75	3.00	*

* Variables included in the multiple correspondence analysis (MCA).

**Table 4:** Characteristics of the scope of use of local resources in feeding livestock on pig farms in communities of Jipijapa (N = 55).

Variable	Class	Absolute Frequency	Relative Frequency %	MCA *
Feed sources	Commercial food	0	0.00	*
	Farm food	30	54.5	
	Both	25	45.4	
Farm food	Corn, banana, cassava, kitchen waste	1	1.8	*
	Corn, banana, kitchen waste	3	5.5	
	Corn, cassava, banana, tagua, kitchen waste	7	12.7	
	Corn, banana, cassava	3	5.5	
	Corn, banana, cassava, ivory palm, kitchen waste, pumpkin	9	16.4	
	Corn, banana, cassava, pumpkin, kitchen waste	2	3.6	
	Corn, banana, cassava, pumpkin, kitchen waste	8	14.5	
	Corn, cassava, banana, plantain, pumpkin	6	10.9	
	Cassava, banana	2	3.6	
	Corn, kitchen waste	1	1.8	
	Banana, kitchen waste	1	1.8	
	Corn, cassava, kitchen waste	1	1.8	
	Corn, banana	3	5.5	
	Banana, ivory palm, bean	1	1.8	
	Corn, cassava, banana, ivory palm	5	9.1	
	Banana, rice powder	2	3.6	
Farm food preparation	Cooked	19	34.5	
	Cut up	28	50.9	
	Cooked and cut up	7	12.7	
	Milled	1	1.8	
Sowing food crops ^1^	Yes/No	33	60.0	
Has food preparation equipment ^1^	Yes/No	0	0.0	
Would try new food alternatives ^1^	Yes/No	52	94.5	

¹ Represents the number of affirmative answers. * Variables used in multiple correspondence analysis (MCA).

**Table 5:** Factors of multiple correspondence analysis.

	Factors ^1^		
	F1	F2	F3
Age	0.03	0.17	0.15
Schooling	0.26	0.22	0.28
Occupation	0.22	**0.47**	0.29
Basic services	**0.62**	0.20	0.02
Drinking water supply	**0.58**	0.14	0.03
Waste operation	**0.60**	0.43	0.19
Common diseases	0.47	0.09	0.16
Production system	0.45	0.08	0.22
Pigsty construction material	0.25	**0.64**	0.13
Pig breed	**0.55**	0.37	0.10
Supply practices	0.08	0.05	**0.58**
Age of the start of fattening	0.27	0.26	**0.50**
Fattening duration	0.19	**0.58**	0.23
Breeding	0.19	0.34	0.01
Slaughtered sale price	**0.57**	0.28	0.40
Practice plan and record	0.14	**0.40**	0.17
Food sources	0.37	0.35	0.06
Farm food	0.36	**0.51**	0.12
Total active	6.19	5.56	3.64
% of variance	34.40	30.90	20.22

^1^ In bold variables with high weight in the variation of each factor.

**Table 6:** Locations of farms of small producers of Jipijapa pigs in each identified group (% over the total of farms in the group). In brackets is the number of farms.

Location	G1 (12)	G2 (12)	G3 (12)	G4 (9)	G5 (10)
Colón Alfaro	0	0	0	0	100.00
Albajacal	83.33	0	0	0	0
La Susana	0	25.00	0	33.33	0
Flor del Salto	0	0	0	66.67	0
La Cuesta	0	8.33	100.00	0	0
Santa Lucia	8.33	33.33	0	0	0
El Páramo	8.33	33.33	0	0	0

**Table 7:** Characteristics and comparative analysis of the five groups of small producers of backyard pigs in Jipijapa. In brackets is the number of farms.

		G1 (12)	G2 (12)	G3 (12)	G4 (9)	G5 (10)	Total (55)	*p*-Value
Socioeconomic scope	Male gender (%)	75.00	50.00	75.00	88.89	80.00	72.73	ns
	Middle age (years)	66.67 ^b^ ± 14.08	51.75 ^ab^ ± 17.62	44.33 ^a^ ± 19.11	54.33 ^ab^ ± 11.76	47.10 ^ab^ ± 18.95	53.31 ± 17.52	ns
	Married (%)	33.33 ^b^	66.67 ^ab^	41.67 ^b^	100.00 ^a^	70.00 ^ab^	60.00	**
	Have children (%)	83.33	58.33	58.33	77.78	60.00	67.27	ns
	Wife or children dedicated to raising pigs (%)	83.33	83.33	66.67	100.00	90.00	83.64	ns
	Completed primary school (%)	75.00	83.33	75.00	88.89	90.00	81.82	ns
	Completed secondary school (%)	16.67 ^b^	33.33 ^ab^	16.67 ^b^	33.33 ^ab^	70.00 ^a^	32.73	*
	Farmer on own farm (%)	0.00 ^d^	16.67 ^cd^	41.67 ^bc^	88.89 ^a^	60.00 ^ab^	38.18	***
	Farm has piped running water (%)	25.00 ^c^	58.33 ^b^	0.00 ^c^	22.22 ^c^	100.00 ^a^	40.00	***
	Has electricity (%)	83.33 ^a^	41.67 ^b^	100.00 ^a^	100.00 ^a^	100.00 ^a^	83.64	***
	Has drinking water (%)	0.00 ^c^	0.00 ^c^	0.00 ^c^	22.22 ^b^	100.00 ^a^	21.82	***
	Has garbage collection service (%)	0.00 ^c^	0.00 ^c^	0.00 ^c^	33.33 ^c^	90.00 ^a^	21.82	***
	Belongs to an association (%)	66.67 ^a^	16.67 ^c^	8.33 ^c^	55.56 ^ab^	20.00 ^bc^	32.73	**
	Has received professional training (%)	8.33	8.33	8.33	22.22	30.00	14.55	ns
	Produces only for self-consumption (%)	58.33 ^a^	41.67 ^a^	0.00 ^a^	22.22 ^ab^	60.00 ^a^	36.36	**
Production systems	Grazing practice (%)	33.33 ^b^	25.00 ^cb^	100.00 ^a^	100.00 ^a^	0.00 ^c^	58.33	***
	Average number of pigs	3.33 ± 3.26	5.08 ± 3.87	5.33 ± 4.35	5.00 ± 2.96	3.40 ± 5.85	4.44 ± 4.11	ns
	Has a traditional pigsty (%)	8.33 ^c^	58.33 ^ab^	91.67 ^b^	66.67 ^ab^	33.33 ^ac^	54.17	***
	Breeds Creole pigs (%)	100.00 ^a^	66.67 ^ab^	41.67 ^b^	66.67 ^ab^	66.67 ^b^	68.75	*
	Provides water ad libitum (%)	33.33 ^ab^	8.33 ^ab^	0.00 ^b^	44.44 ^a^	33.33 ^a^	20.83	*
	Provides food ad libitum (%)	33.33 ^a^	0.00 ^b^	0.00 ^b^	0.00 ^b^	33.33 ^b^	10.42	*
	Castration (%)	83.33 ^a^	75.00 ^a^	8.33 ^b^	44.44 ^ab^	66.67 ^a^	54.17	***
	Draining (%)	100.00 ^a^	83.33 ^ab^	100.00 ^a^	66.67 ^b^	100.00 ^a^	89.58	*
	Deworming (%)	100.00 ^a^	83.33 ^ab^	100.00 ^a^	66.67 ^b^	100.00 ^a^	89.59	*
	Planned reproduction (%)	16.67	8.33	33.33	11.11	100.00	22.92	*
	Age when pigs start fattening (months)	2.25 ^ab^ ± 0.45	2.67 ^b^ ± 0.98	3.58 ^a^ ± 0.79	1.44 ^c^ ± 0.53	2.10 ^ab^ ± 0.74	2.47 ± 0.10	***
	Weight when pigs start fattening (lb)	15.42 ^c^ ± 2.57	19.17 ^c^ ± 7.64	52.50 ^a^ ± 11.38	18.33 ^c^ ± 3.53	29.40 ^b^ ± 6.85	27.36 ± 15.84	***
	Age when they end fattening (months)	12.83 ^a^ ± 2.48	9.25 ^b^ ± 2.30	8.00 ^b^ ± 1.59	8.44 ^b^ ± 1.74	8.70 ^b^ ± 1.95	9.53 ± 2.69	***
	Weight when pigs finish fattening (lb)	107.50 ± 17.12	115.83 ± 21.93	110.83 ± 23.91	107.22 ± 23.86	140.00 ± 45.95	115.90 ± 29.19	ns
	Keeps record book for breeding (%)	0.00	16.67	8.33	33.33	33.33	14.58	ns
	Price per sale alive (USD/lb)	1.44 ^ab^ ± 0.20	1.55 ^a^ ± 0.33	1.50 ^a^ ± 0.00	1.53 ^a^ ± 0.10	1.23 ^b^ ± 0.09	1.45 ± 0.22	**
	Price per sale slaughtered (USD/lb)	2.50 ^b^ ± 0.00	2.49 ^b^ ± 0.27	2.75 ^a^ ± 0.00	2.89 ^a^ ± 0.22	2.27 ^b^ ± 0.30	2.57 ± 0.28	***
Use of local resources	Uses farm food as an alternative (%)	66.67 ^ab^	58.33 ^ab^	25.00 ^b^	33.33 ^b^	90.00 ^a^	54.55	*
	Average number of alternatives used	6.00 ^a^ ± 1.04	5.00 ^ab^ ± 1.28	4.50 ^b^ ± 1.44	4.33 ^b^ ± 1.12	2.50 ^c^ ± 0.53	4.54 ± 1.58	***
	Grows specifically for livestock (%)	16.67 ^b^	50.00 ^b^	91.67 ^a^	100.00 ^a^	50.00 ^b^	60.00	***
	Uses kitchen waste (%)	75.00 ^ab^	58.33 ^ab^	33.33 ^b^	88.89 ^a^	50.00 ^ab^	60.00	*
	Uses banana as alternative (%)	83.33 ^a^	83.33 ^a^	75.00 ^a^	0.00 ^b^	0.00 ^b^	52.73	***
	Uses ivory palm as alternative (%)	58.33 ^a^	41.67 ^a^	33.33 ^ab^	66.67 ^a^	0.00 ^b^	40.00	**
	Uses pumpkin as alternative (%)	83.33 ^a^	33.33 ^b^	25.00 ^b^	0.00 ^b^	0.00 ^b^	30.91	***
	Cooks food (%)	66.67 ^ab^	66.60 ^ab^	0.00 ^c^	77.78 ^a^	30.00 ^bc^	47.27	***
	Only cuts up food (%)	33.33 ^b^	33.33 ^b^	100.00 ^a^	11.11 ^b^	30.00 ^b^	43.64	***

* *p* < 0.05; ** *p* < 0.01; *** *p* < 0.001; ns, no significant difference. ^a, b, c^ superscript letters indicate significative differences amongst species (*p* < 0.05).

## Data Availability

This is not applicable, as the data are not in any data repository with public access. However, if an editorial committee needs access, we will happily provide them with it; please use this email: pa1rosee@uco.es.
